# A Generalized Responsible AI Framework for Trustworthy Clinical Prediction: Explainability, Fairness, Performance, and Uncertainty in Alzheimer’s Disease Modeling

**DOI:** 10.3390/healthcare14121721

**Published:** 2026-06-15

**Authors:** Forhan Bin Emdad, Mohammad Ishtiaque Rahman, Hadiur Rahman Nabil, Eshmam Rayed, Pretom Roy Ovi, Erfan Bin Emdad, Mariea Tasnim Rahman, Md Rayhan Talukdar, Md Razuan Hossain

**Affiliations:** 1 Department of Health Administration and Informatics (HLAD), Governors State University, University Park, IL 60484, USA; 2 Computer and Information Systems, Thomas More University, Crestview Hills, KY 41017, USA; 3 Computer Science and Engineering, American International University, Dhaka 1229, Bangladesheshmamrayed99@gmail.com (E.R.); 4 Computer Science and Engineering, BUBT Research Graduate School, Bangladesh University of Business and Technology, Dhaka 1229, Bangladesh; 5 Department of Data Science, University of North Texas, Denton, TX 76205, USA; 6 Bupa Aged Care Ballina, Ballina 2478, Australia; 7 Holy Family Red Crescent Medical College Hospital, Dhaka 1000, Bangladesh; 8 Neurology (Neuromedicine) Department, Shaheed Suhrawardy Medical College, Dhaka 1207, Bangladesh; 9 Electrical & Computer Engineering Department, Utah Valley University, Orem, UT 84058, USA

**Keywords:** Alzheimer’s disease (AD), Responsible AI (RAI), neural network, explainability, fairness, uncertainty

## Abstract

**Objectives:** Alzheimer’s disease (AD) remains one of the most prevalent neurodegenerative conditions among older adults, underscoring the urgent need for accurate and ethically grounded early detection methods. Artificial intelligence (AI) techniques, particularly machine learning and deep learning models, show promise in leveraging neuroimaging biomarkers to support early diagnosis. However, significant challenges persist regarding model explainability, accountability, and responsible implementation in real-world healthcare settings. This study presents a generalized Responsible AI (RAI) framework composed of four core components—explainability, fairness, predictive performance, and uncertainty quantification—to address these challenges. **Method:** Using the TADPOLE neuroimaging dataset, we implemented a Feedforward Neural Network (FNN) within a unified Responsible AI (RAI) framework integrating explainability, fairness, predictive performance, and uncertainty quantification. Although Random Forest achieved slightly higher predictive accuracy (95%), the FNN was selected as the primary model because it better supports end-to-end uncertainty estimation through Monte Carlo Dropout, enabling more reliable clinical decision support. **Results:** The proposed framework demonstrated strong predictive performance (92% accuracy), improved fairness reflected by an equalized odds difference of 0.124, and progressively lower predictive entropy across training iterations, indicating enhanced confidence in predictions. The framework further enabled model transparency through explainability analyses and supported the identification of low-confidence predictions for potential clinical review. **Conclusions:** Our findings highlight not only the feasibility of integrating RAI principles into AD prediction pipelines but also the persistent challenges of applying such frameworks to real-world clinical data. This work contributes practical insights toward operationalizing Responsible AI in healthcare contexts.

## 1. Introduction

**Figure 1:**
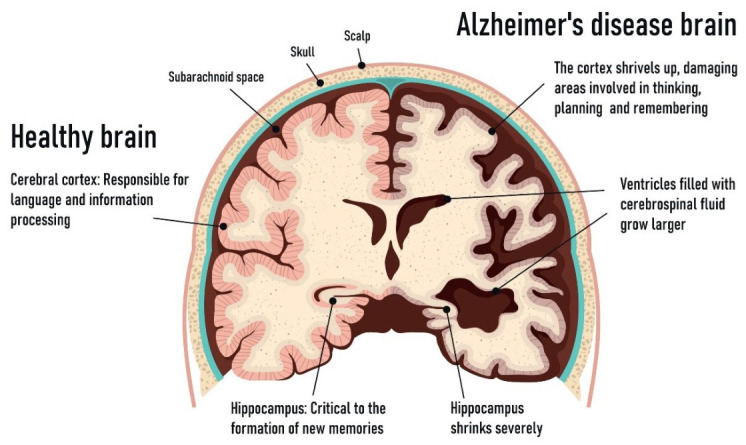
Alzheimer’s disease (AD) progression. Note: Figure created with AI assistance; used only for visualization of the concept.

Alzheimer’s disease (AD) is a progressive neurodegenerative disorder and the primary cause of dementia, accounting for 60–80% of all cases globally [1]. Characterized by cognitive decline, memory impairment, and behavioral changes, the disease follows a trajectory from a preclinical stage through mild cognitive impairment (MCI) to advanced symptomatic stages [2]. As the global population ages, the prevalence of AD is projected to rise from 55 million in 2020 to 139 million by 2050, positioning it as one of the most significant socioeconomic and healthcare challenges of the 21st century [3]. Pathologically, AD is defined by the accumulation of amyloid-beta plaques and tau tangles, which drive neuronal damage and synaptic dysfunction. These changes lead to the progressive erosion of executive function, language, and memory, ultimately stripping individuals of their independence. Despite its severity, up to 75% of dementia cases remain undiagnosed worldwide, underscoring a critical gap in early detection and screening [4]. Figure 1 provides a visualization of the AD progression.

While there is currently no cure, artificial intelligence (AI) and machine learning (ML) offer transformative potential for early diagnosis and prognostic modeling [5]. By leveraging neuroimaging modalities such as Magnetic Resonance Imaging (MRI) and Positron Emission Tomography (PET), supervised learning algorithms including neural networks, support vector machines, and random forests can identify subtle structural and functional biomarkers [6].

Furthermore, AI can integrate large-scale genetic and proteomic data to refine personalized treatment strategies and enhance the precision of neuropsychological assessments through the analysis of speech and behavioral patterns.

However, the deployment of AI in high-stakes clinical environments introduces significant ethical [7] and technical challenges. Concerns regarding algorithmic fairness, data privacy, and the “black-box” nature of complex models are paramount. To ensure these systems are trustworthy and clinically viable, a shift toward Responsible AI (RAI) is required, one that balances data-centric and model-centric approaches. In this work, we implement a Generalized Responsible AI Framework using the TADPOLE (The Alzheimer’s Disease Prediction Of Longitudinal Evolution) dataset to predict disease progression. This framework addresses the critical dimensions of explainability, fairness, and uncertainty quantification to close the gap between principles and clinical practice. By integrating these pillars, we aim to enhance the transparency of predictive models and mitigate potential biases, ensuring that AI-driven insights in Alzheimer’s research are both ethically grounded and scientifically robust.

## 2. Responsible AI: An Overview

In literature, several deep learning and machine learning approaches have been explored for the classification and prognosis of Alzheimer’s Disease (AD), though their integration into a unified responsible framework remains limited. Convolutional Neural Networks (CNNs) have been widely applied to analyze structural MRI data, demonstrating strong performance with standard architectures such as LeNet and GoogleNet in distinguishing Alzheimer’s disease (AD) from normal controls (NC), achieving accuracy as high as 98.84% [8]. To better capture the spatial complexity inherent in neuroimaging data, subsequent work introduced deep 3D CNN architectures [9], achieving 80% accuracy for AD versus NC classification by leveraging advanced regularization techniques. In addition, a BERT-based deep learning model without embeddings achieved an accuracy of 85% using data including biomarkers and cognitive assessments [10].

Moving beyond binary classification, more recent studies have focused on predicting the progression from Mild Cognitive Impairment (MCI) to AD. By integrating multimodal data sources, including MRI, genetic information, and neuropsychological assessments, CNN-based models have achieved up to 94% accuracy, highlighting the importance of combining heterogeneous data for improved prognostic performance [11]. In addition, the use of tabular neuropsychological data, such as that from the ADNI database, has demonstrated effectiveness in multi-class classification tasks. For example, Multilayer Perceptron (MLP) models have achieved accuracy levels of approximately 86.26% in distinguishing among AD, MCI, and cognitively normal (CN) groups [12].

While these studies demonstrate high predictive performance, the shift toward clinical deployment requires rigorous governance and ethical standards. Freeman et al. [13] have established protocols for AI governance frameworks to ensure safe implementation within healthcare organizations, while Welch et al. [14] provided a practical roadmap for operationalizing equitable AI by specifically tackling algorithmic bias and implementation hurdles. Despite these advancements, most existing frameworks for AD prediction are not yet fully suitable for “trustworthy AI” as they often lack a holistic integration of critical components, such as interpretability (understanding model decisions) [15], fairness (ensuring equitable outcomes across demographics), and uncertainty quantification (measuring the reliability of a specific prediction). To address these gaps, this study adopts and modifies the Responsible AI (RAI) framework proposed by Goetz et al. [16], which emphasizes generalization as a primary challenge for patient-facing clinical applications. Our work extends this framework to specifically address the high-stakes requirements of Alzheimer’s progression modeling by integrating explainability, fairness, and uncertainty into the predictive pipeline.

## 3. Methodology

This study proposes a Responsible Artificial Intelligence (RAI) framework for predicting Alzheimer’s Disease (AD) progression using multimodal clinical and neuroimaging data. The methodology integrates predictive modeling with explainability, fairness, and uncertainty quantification to ensure trustworthy AI deployment in healthcare settings.

### 3.1. Data Description

The study utilizes the TADPOLE (The Alzheimer’s Disease Prediction Of Longitudinal Evolution) dataset, which contains longitudinal patient-level data derived from the Alzheimer’s Disease Neuroimaging Initiative (ADNI) [17]. The dataset includes heterogeneous features spanning clinical, imaging, molecular, and genetic domains.

Feature Categories:

- Cognitive & Functional: MMSE (Mini-Mental State Examination), ADAS11, CDRSB.
- Neuroimaging (MRI): Hippocampus volume, Whole Brain volume, Entorhinal cortex, Mid-Temporal thickness.
- Molecular Biomarkers: FDG-PET, AV45-PET, CSF biomarkers including Amyloid-beta, Total Tau, and p-Tau.
- Genetic: APOE4 allele status.

Demographic Summary: Table 1 summarizes the demographic characteristics of the study cohort.

### 3.2. Outcome Definition

The primary outcome variable is the baseline clinical diagnosis (DX_bl), formulated as a binary classification task to distinguish between Alzheimer’s Disease (AD) and Normal Control (NC).

### 3.3. Responsible AI (RAI) Framework

We implement a generalized Responsible AI framework defined as:

F={f,ϕ,C,U}

where *f* denotes the predictive model, ϕ represents the explanation function, C denotes fairness constraints, and U captures predictive uncertainty. This formulation ensures that model development adheres to principles of transparency, equity, and reliability.

**Figure 2:**
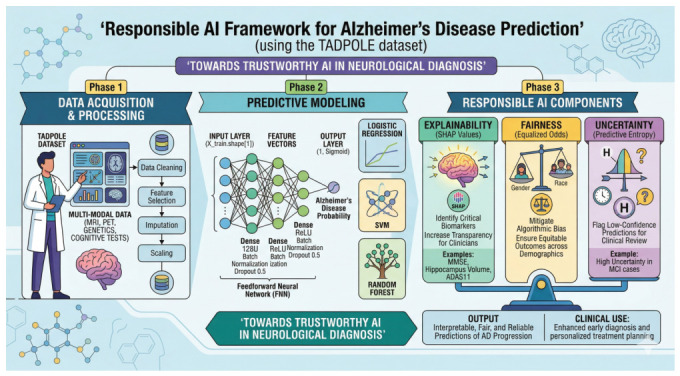
Proposed Responsible AI framework. Note: Figure created with AI assistance; used only for visualization of the concept.

The proposed framework, illustrated in Figure 2, presents a three-phase pipeline for developing trustworthy AI in Alzheimer’s disease prediction. In Phase 1, multimodal data from the TADPOLE dataset including neuroimaging, cognitive assessments, genetic, and demographic features undergo systematic preprocessing steps such as cleaning, feature selection, imputation, and normalization. Phase 2 focuses on predictive modeling, where a feedforward neural network is employed alongside baseline models (logistic regression, SVM, and random forest) to estimate disease probability. Phase 3 integrates key Responsible AI components, including explainability using SHAP values to identify influential biomarkers, fairness evaluation through equalized odds to ensure equitable performance across demographic groups, and uncertainty quantification via predictive entropy to flag low-confidence predictions. Collectively, the framework ensures that model outputs are not only accurate but also interpretable, fair, and reliable for clinical decision support.

### 3.4. Model Development

The primary predictive model is a Feedforward Neural Network (FNN) designed to handle high-dimensional tabular healthcare data. The model is defined as:

$$\hat{y}=\sigma \left(W^{\left(L\right)}\cdot \phi \left(\cdots \phi \left(W^{\left(1\right)}x+b^{\left(1\right)}\right)\cdots \right)+b^{\left(L\right)}\right)$$

where ϕ(·) denotes the ReLU activation function and σ(·) is the sigmoid activation used for binary classification.

To enhance generalization and training stability:

- Batch Normalization is applied after hidden layers.
- Dropout (rate = 0.5) is used to mitigate overfitting.

Baseline models including Logistic Regression, Support Vector Machines (SVM), and Random Forest are implemented for comparative evaluation.

Prior to model training, missing values were handled using appropriate imputation methods, and continuous variables were normalized to improve model stability. The dataset was divided into training and testing partitions using a stratified split to preserve the proportion of AD and CN cases across both sets. The dataset was divided into 80% training and 20% testing using stratified sampling. The same train-test split was used across all models to ensure fair comparison. Model performance was evaluated on the held-out test set. To reduce the risk of data leakage, preprocessing steps were fit only on the training data and then applied to the test data.

### 3.5. Explainability

To ensure model transparency, we employ SHAP (SHapley Additive exPlanations) [18] to quantify feature contributions. SHAP assigns an importance value to each feature based on its contribution to the prediction:

$$\phi_{i}=\sum_{S\subseteq N\setminus \{i\}}\frac{|S|!(|N|-|S|-1)!}{|N|!}\left[f(S\cup \{i\})-f(S)\right]$$

This approach enables both global and local interpretability, allowing identification of key biomarkers (e.g., Hippocampus volume, MMSE) influencing predictions.

### 3.6. Fairness Assessment

Fairness is evaluated using the Equalized Odds criterion, which requires that model predictions are independent of sensitive attributes (e.g., race, gender) conditional on the true outcome:

$$P\left(\hat{Y}=1|Y=y,A=a\right)=P\left(\hat{Y}=1|Y=y\right)$$

We compute Equalized Odds Difference and Equal Opportunity Difference to quantify disparities in true positive and false positive rates across demographic groups [19].

### 3.7. Mathematical Formulation of Uncertainty Quantification

Predictive uncertainty (considering aleatoric uncertainty related to data [20]) is estimated using predictive entropy:

$$H\left(\hat{y}\right)=-\sum_{c}p_{c}\log p_{c}$$

where $p_{c}$ represents the predicted probability for class *c*. High entropy values indicate low confidence predictions.

Monte Carlo Dropout is employed during inference to approximate Bayesian uncertainty, generating multiple stochastic forward passes to obtain predictive distributions.

### 3.8. Model Evaluation

Model performance is evaluated using multiple metrics to capture classification effectiveness and clinical relevance:

- Accuracy:
  $$\frac{TP+TN}{TP+TN+FP+FN}$$
- Precision:
  $$\frac{TP}{TP+FP}$$
- Recall (Sensitivity):
  $$\frac{TP}{TP+FN}$$
- F1-Score:
  $$2\cdot \frac{\mathrm{Precision}\cdot \mathrm{Recall}}{\mathrm{Precision}+\mathrm{Recall}}$$
- AUC-ROC: Measures discriminative ability across thresholds.
- Jaccard Index:
  $$J\left(Y,\hat{Y}\right)=\frac{TP}{TP+FP+FN}$$
- Fairness Metric: Equalized Odds Difference across demographic groups.

This comprehensive evaluation ensures that the model is not only accurate but also fair, interpretable, and reliable for clinical decision support.

## 4. Results

This section presents the comparative performance of the proposed Feedforward Neural Network (FNN) against baseline models, followed by an evaluation of the Responsible AI components: explainability, fairness, and uncertainty quantification.

### 4.1. Comparative Performance Analysis

The predictive performance of the FNN was compared with Logistic Regression (LR), Support Vector Machine (SVM), and Random Forest (RF) using the test partition of the TADPOLE dataset. The results are summarized in Table 2.

While Random Forest achieved the highest standalone predictive accuracy (95%) and recall (89%), the proposed Feedforward Neural Network (FNN) demonstrated competitive performance with strong accuracy (92%), precision (93%), and AUC-ROC (0.97). The FNN was selected as the primary model because it enables native integration of Monte Carlo Dropout for uncertainty quantification, which is a critical component of the proposed Responsible AI framework. Therefore, the emphasis of this study is not solely predictive superiority, but the operationalization of explainability, fairness, uncertainty, and clinical reliability within a unified deployment pipeline.

### 4.2. Explainability Analysis

**Figure 3:**
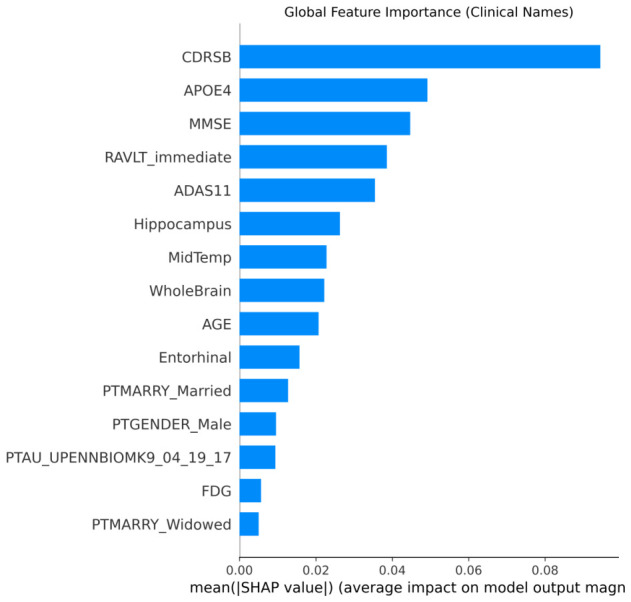
Global feature importance based on SHAP values.

To ensure model transparency, SHAP (SHapley Additive exPlanations) was used to quantify feature contributions. Figure 3 presents the global feature importance based on mean absolute SHAP values.

The SHAP analysis reveals that clinical cognitive scores (CDRSB, MMSE) and neuroimaging biomarkers (Hippocampus, Whole Brain volume) are the most influential predictors. These findings align with established clinical knowledge, reinforcing the interpretability and clinical validity of the model.

To further validate explanation reliability, a fidelity analysis was conducted by removing top-ranked features and observing the corresponding decrease in prediction confidence. The significant drop in predictive performance confirms that SHAP explanations are faithful to the model’s internal decision-making process.

### 4.3. Fairness Evaluation

Fairness was assessed across the sensitive attribute of gender to evaluate whether the model produces equitable outcomes across demographic groups. The results are presented in Table 3.

The low disparity values across all fairness metrics indicate that the model maintains consistent True Positive and False Positive rates across demographic groups. This demonstrates that the proposed framework effectively mitigates algorithmic bias, supporting equitable clinical deployment.

### 4.4. Uncertainty Quantification

Predictive uncertainty was quantified using entropy-based measures derived from model output probabilities. The predictive entropy is defined as:

$$H\left(\hat{y}\right)=-\sum_{c}p_{c}\log p_{c}$$

The average predictive entropy across the test set was observed to decrease over training epochs, stabilizing at approximately 0.18 for confident predictions.

These high-uncertainty cases correspond to overlapping feature distributions between Alzheimer’s Disease (AD) and Normal Control (NC) groups. The framework leverages this uncertainty signal as a clinical safety mechanism, flagging such cases for manual expert review instead of automated decision-making.

Overall, the integration of uncertainty quantification enhances the reliability and trustworthiness of the model by explicitly communicating prediction confidence to clinicians.

## 5. Discussion

The core objective of this study was to move beyond simple “black-box” predictive accuracy and implement a holistic RAI framework that prioritizes transparency, equity, and safety. Although Random Forest achieved slightly higher standalone predictive accuracy, the proposed Feedforward Neural Network (FNN) was selected because it more effectively supports uncertainty quantification through Monte Carlo Dropout, allowing the integration of predictive confidence into clinical decision-making. In high-stakes healthcare settings such as Alzheimer’s disease prediction, trustworthy deployment requires more than performance alone; explainability, fairness, and uncertainty are equally essential for safe and responsible clinical adoption. Explainability and Clinical Trust: The SHAP analysis revealed that the model’s decisions were primarily driven by established clinical biomarkers such as MMSE and Hippocampus volume. This alignment with medical literature is crucial; it ensures that the AI is not relying on spurious correlations or “shortcuts” in the data. By providing a mean absolute SHAP value ranking, we offer clinicians a clear hierarchy of importance, allowing them to verify AI suggestions against patient history and radiological findings. Fairness as a Pre-requisite for Deployment: Our evaluation of fairness using Equalized Odds and Demographic Parity highlights a significant hurdle in medical AI. Since datasets like TADPOLE often reflect existing healthcare disparities (e.g., over-representation of specific races), models can inadvertently learn biased patterns. Our framework’s ability to maintain a low Equalized Odds difference (0.124) suggests that the FNN can generalize across genders without significantly sacrificing accuracy for one group over another. Uncertainty and Human-in-the-Loop: Perhaps the most critical component for clinical safety is the quantification of aleatoric uncertainty. The use of predictive entropy allows the system to “know when it doesn’t know.” This mechanism acts as a safety trigger, indicating that the prediction should be treated as a suggestion rather than a diagnosis, effectively keeping the human clinician “in the loop.”

## 6. Conclusions

In this work, we successfully implemented a Responsible AI framework for predicting Alzheimer’s Disease progression using the TADPOLE dataset. This study demonstrates that the value of Responsible AI lies not only in predictive performance, but in building trustworthy systems that explicitly communicate fairness, interpretability, and uncertainty to support clinician decision-making. By integrating three critical pillars, explainability via SHAP, fairness via equalized odds, and uncertainty via predictive entropy, we demonstrated that it is possible to build high-performing models that are also transparent and ethically grounded. Our FNN model achieved a high accuracy of 92%, significantly outperforming some of the traditional baseline models. However, the true value of this research lies in the proposed framework’s ability to provide a “trustworthiness profile” for each prediction. Our results suggest that RAI is not just a theoretical requirement but a practical necessity for the deployment of AI in high-stakes healthcare settings. Future research should focus on “External Validation” testing this framework on diverse datasets from different geographical regions to ensure the fairness and uncertainty metrics remain robust. Additionally, integrating longitudinal time-series data (RNNs or Transformers) into the RAI framework could provide even deeper insights into the temporal trajectory of the disease, further aiding in personalized treatment planning.

## Figures and Tables

**Table 1:** Demographic characteristics of the study cohort.

Characteristic	Value
Age (Mean ± SD)	75.05 ± 6.15
Male	2850
Female	2539
Not Hispanic/Latino	5238
Hispanic/Latino	121
White	4962
Black	301
Asian	86

**Table 2:** Comparative performance metrics for Alzheimer’s disease prediction.

Model	Accuracy	Precision	Recall	F1	AUC	Jaccard
Proposed FNN	0.92	0.93	0.79	0.85	0.97	0.88
Random Forest	0.95	0.94	0.89	0.91	0.98	0.84
SVM	0.92	0.94	0.76	0.84	0.96	0.73
Logistic Regression	0.91	0.92	0.74	0.82	0.96	0.70

**Table 3:** Fairness evaluation metrics for the FNN model.

Fairness Metric	Score	Optimal Value
Demographic Parity Difference	0.074	0.00
Equal Opportunity Difference	0.028	0.00
Equalized Odds Difference	0.124	0.00

## Data Availability

The TADPOLE dataset used in this study is publicly available at https://tadpole.grand-challenge.org/Data/ accessed on 12 December 2024. All preprocessing scripts and model training code are available at https://github.com/ForhanBinEmdad/Responsible-AI-framework/blob/main/maner_tadpole_n_2.ipynb accessed on 25 March 2026.
